# Human steroidogenesis: implications for controlled ovarian stimulation with exogenous gonadotropins

**DOI:** 10.1186/1477-7827-12-128

**Published:** 2014-12-28

**Authors:** Claus Y Andersen, Diego Ezcurra

**Affiliations:** Laboratory of Reproductive Biology, University Hospital of Copenhagen, Copenhagen, Denmark; Global Medical Affairs, EMD/Merck Serono, One Technology Place, Rockland, MA 02370 USA

**Keywords:** Controlled ovarian stimulation, Progesterone elevation, Steroidogenesis, Gonadotropins, Luteinising hormone

## Abstract

In the menstrual cycle, the mid-cycle surge of gonadotropins (both luteinising hormone [LH] and follicle-stimulating hormone [FSH]) signals the initiation of the periovulatory interval, during which the follicle augments progesterone production and begins to luteinise, ultimately leading to the rupture of the follicle wall and the release of an oocyte. The administration of gonadotropins in controlled ovarian stimulation (COS) leads to supraphysiological steroid concentrations of a very different profile compared with those seen during natural cycles. It has been suggested that these high steroid concentrations cause alterations in endometrial development, affecting oocyte viability in assisted reproductive technology. Furthermore, it has been proposed that elevated progesterone levels have a negative effect on the reproductive outcome of COS. This may arise from an asynchrony between embryo stage and endometrium status at the window of implantation. The regulation of progesterone production by the developing follicles during COS is a complicated interplay of hormonal systems involving the theca and granulosa cells, and the effect of the actions of both LH and FSH. The present paper reviews current knowledge of the regulation of progesterone in the human ovary during the follicular phase and highlights areas where knowledge remains limited. In this review, we provide in-depth information outlining the regulation and function of gonadotropins in the complicated area of steroidogenesis. Based on current evidence, it is not clear whether the high levels of progesterone produced during COS have detrimental effects on fertility.

## Background

Controlled ovarian stimulation (COS) is an important component of assisted reproductive technology (ART). Following exogenous gonadotropin administration, COS leads to the development of multiple ovarian follicles. It has been suggested that due to endocrine effects on the endometrium COS may have a deleterious effect on the window of implantation during *in vitro* fertilisation (IVF) cycles. Based on post-ovulation histological assessments, Bourgain and Devroey [1] suggested that COS is associated with ‘advancement of endometrial development’. More recent studies have also shown differences in gene expression; however, the differences suggest that endometrial development is delayed by COS, and that this has clinical implications [2]. Although the clinical significance of these effects is still not clearly defined, and may involve only a relatively small proportion of patients, it is important to determine whether endometrial development is affected by the supraphysiological levels of steroids produced by multiple preovulatory follicles as a result of COS. It is also important to determine the precise nature of the milieu of circulating steroids that are present during COS.

This review provides details on the complex area of human ovarian steroidogenesis during the follicular phase at the molecular, functional and clinical level, and provides evidence to address the question of whether high progesterone levels produced during the late follicular phase have an effect on pregnancy outcomes following COS.

### Human ovarian steroidogenesis

#### Gonadotropins in steroidogenesis

The development of human ovarian follicles depends on the sequential effects of the two principal gonadotropins, follicle-stimulating hormone (FSH) and luteinising hormone (LH). FSH acts on early antral follicles to stimulate growth (cell division), steroidogenesis, and the expression of LH receptors. Subsequently, LH, in synergy with FSH, acts on the FSH-stimulated follicle to maintain growth, and is eventually responsible for the processes of luteinisation and ovulation. In the normal cycle, the dominant follicle maintains its growth and hormone output in the face of declining FSH concentrations by the acquisition of increased sensitivity to LH [3, 4]. Through the follicular stage, FSH and LH both stimulate cyclic adenosine monophosphate (cAMP) production and elicit growth and oestrogen secretion through this same mechanism [5, 6].

The implementation of COS using exogenous gonadotropins leads to supraphysiological levels of circulating FSH throughout the follicular phase. These levels are higher and of a very different pattern compared with that seen during the normal menstrual cycle [7, 8].

Activation of LH and FSH receptors on granulosa cells leads to the activation of most known intracellular signalling pathways, i.e. the adenylate cyclase, protein kinase C (inositol phosphate), the mitogen-activated protein (MAP) kinase, and extracellular signal-regulated kinase pathways [5]. In human theca cells, signalling pathways are unknown; however, data from studies with bovine theca cells indicate that LH-induced Akt phosphorylation is modulated via the MAP kinase signalling pathway [9]. FSH-induced granulosa cell differentiation also leads to induction of LH receptor expression, increased steroidogenesis, and induction of various transforming growth factor beta (TGF-b)-related growth factors. While this is reasonably well understood in natural cycles of laboratory animals and, partially, in humans, less is known about how these processes are affected by the supraphysiological concentrations of FSH used in COS, and how this might in turn affect oocyte viability in ART.

#### Modification of responsiveness of granulosa cells to LH

In the normal cycle the dominant follicle continues to grow in the environment of falling FSH concentrations by promoting its sensitivity to gonadotrophins. This includes the establishment of granulosa-cell sensitivity to LH. The granulosa cells in small antral follicles show little sensitivity to LH; however, as they grow, the granulosa cells become highly sensitive to LH and in humans the sensitivity towards FSH becomes reduced [4]. It is through this mechanism that luteinisation takes place in response to the LH surge.

Activation of the LH receptor in granulosa cells leads to the transcription and protein production of two key enzymes of steroidogenesis: cholesterol side-chain cleavage (P450scc) and aromatase (P450arom). In a study by Yong et al. granulosa cells removed from follicles prior to the LH surge were differentially dependent upon follicular size, and both FSH and LH strongly stimulated P450arom mRNA expression and estradiol production in mature rat granulosa cells. The study also showed that P450scc mRNA expression and progesterone biosynthesis were maximal only in the presence of both FSH and LH [10].

It is likely, therefore, that as follicles mature the role of LH in steroid biosynthesis becomes more important. Not only is LH required for supply of the androgen substrate for aromatase, it is also important in the regulation of the aromatase enzyme itself.

#### The steroidogenic pathway

**Figure 1:**
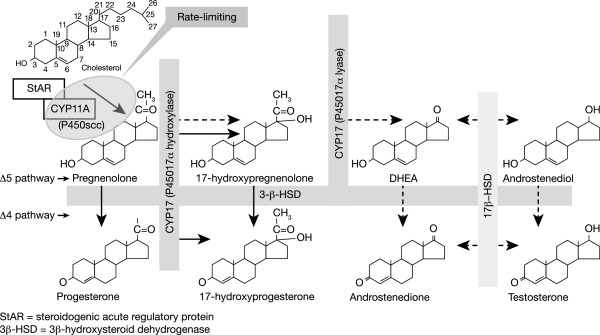
**Human ovarian steroidogenesis.** Dashed arrows show the route of pregnenolone metabolism via the Δ5 pathway. Solid arrows show an alternative route of pregnenolone metabolism beginning with the conversion of pregnenolone to progesterone by the action of 3β-HSD. Adapted from Wickenheisser JK, et al. 2006 [12].

The critical step in steroidogenesis is the initial conversion of cholesterol (a carbon27 [C27] steroid) to pregnenolone (the primary C21 product), from which all other steroids are generated (Figure 1) [11, 12]. The cholesterol side-chain cleavage cytochrome P450 enzyme (P450scc), encoded by the CYP11A gene, catalyses this step and is considered to be the rate-limiting step in steroidogenesis.

Following initial cleavage, the resulting pregnenolone is metabolised in one of three pathways, two of which are illustrated in Figure 1, representing those seen in the ovary. One route of metabolism is via the D5 pathway (Figure 1, red arrows); the first two steps are effected by the same enzyme, CYP17 (P45017a), enzyme complex, which exhibits both hydroxylase and lyase activity (the third pathway for pregnenolone metabolism, the adrenal system, is not reviewed in this article). Hydroxylation of pregnenolone at the C17a position yields 17-hydroxypregnenolone, and subsequent removal of the acetyl group forms the androgen precursor dehydroepiandrosterone (DHEA). Importantly, CYP17 is located exclusively in thecal/interstitial cells (i.e. the extrafollicular compartment of the ovary) [13], whereas CYP19 (aromatase) is expressed only in the granulosa cells (i.e. the intrafollicular compartment) [14]. Thus, the theca cells provide the androgens required by the developing follicles for conversion into oestrogens by the granulosa cells [15].

In the ovary, the second route of metabolism for pregnenolone metabolism (Figure 1, blue arrows) begins with the conversion of pregnenolone to progesterone by the action of 3β-HSD. This conversion is essentially irreversible, as are all the dehydrogenase reactions catalysed by 3β-HSD. Progesterone can then be converted to 17-hydroxyprogesterone by CYP17 (the D4 pathway). In humans, however, 17-hydroxyprogesterone cannot be further metabolised [8]. CYP17 exhibits marked species differences, particularly with respect to the lyase reaction. Thus, 17-hydroxypregnenolone and 17-hydroxyprogesterone are both substrates for rodent and guinea-pig CYP17, while the bovine enzyme shows some selectivity for 17-hydroxyprogesterone, but human CYP17 acts on 17-hydroxypregnenolone almost exclusively. In humans, very little 17-hydroxyprogesterone is converted to androstenedione. Thus, in humans, progesterone and/or 17-hydroxyprogesterone produced by the granulosa cells are the end products of the D4 pathway; the only route for estradiol synthesis in the human ovary is via the D5 pathway. One role of 17-hydroxyprogesterone may be to modulate the biological activity of cortisol, thereby reducing the inflammatory-like reactions associated with ovulation [16]. It would be of interest to know whether there are any conditions in which the substrate specificity of human CYP17 is altered such that the enzyme is able to process 17-hydroxyprogesterone.

Overall, the product of cholesterol side-chain cleavage, pregnenolone (C21), has two further routes of metabolism in the ovary: via the D4 pathway to progesterone (also C21) and the D5 pathway to 17aOH pregnenolone, with further side-chain cleavage to the C19 androgens.

In the context of COS, it is important to consider whether the process of steroidogenesis remains the same during stimulated and natural cycles, and to determine which components of the system could be affected by the supraphysiological concentrations of gonadotropins achieved during COS.

#### CYP17 activity

##### Role of CYP17 in the regulation of adrenal steroidogenesis

CYP17 is also known to have a crucial role in determining the class of steroid produced in human adrenal steroidogenesis. Thus, in the zona glomerulosa, CYP17 is absent, which means that C21 deoxy steroids are produced, the main final product being the mineralocorticoid aldosterone. In the zona fasciculata, where CYP17 hydroxylase activity is present but lyase is absent, C21 17-hydroxy steroids are produced, which means that the main product is the glucocorticoid cortisol. Where both hydroxylase and lyase activity are present (in the zona reticularis), the androgen precursor DHEA is produced in large amounts [11].

The implication of this analysis is that it demonstrates that CYP17 activity can have a major influence on the end products of steroidogenesis.

##### Regulation of CYP17 in theca cells

The hydroxylase and lyase activity of CYP17 are located at the same active site on the enzyme, but the discrimination between the two is regulated post-translationally [11]. Similar to all other microsomal forms of cytochrome P450, CYP17 lyase activity depends on the availability of electrons from reduced nicotinamide adenine dinucleotide phosphate (NADP) [17]. This activity is promoted by serine phosphorylation of CYP17 and the allosteric effect of cytochrome b5, both of which optimise the interaction of CYP17 with its obligatory electron donor, P450 oxidoreductase (POR) [17, 18]. Increasing the ratio of POR to CYP17 also increases the ratio of lyase to hydroxylase activity [11].

**Figure 2:**
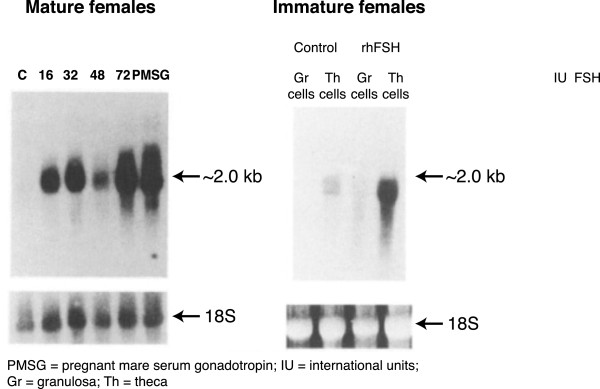
**The effect of r-hFSH administration** ***in vivo*** **on ovarian P450c17α mRNA expression in rats.** FSH values are IU/mL. PMSG: pregnant mare serum gonadotropin; FSH: follicule stimulating hormone; Gr: granulosa; Th: theca. Adapted from Smyth CD, et al. 1993 [13].

In mature female rats, recombinant human FSH (rhFSH) was shown to induce ovarian CYP17 messenger RNA (mRNA) expression of thecal cells (Figure 2, first panel) [13]. In immature females, expression of CYP17 mRNA was undetectable in the absence of endogenous FSH, and rhFSH induced marked expression of CYP17 mRNA in the theca – but not granulosa – cells (Figure 2, second panel) [13]. Therefore, although FSH receptors are expressed only on granulosa cells and CYP17 only on theca calls, FSH appears to be an important regulator of CYP17.

##### Other factors affecting CYP17 activity

Insulin and insulin-like growth factor (IGF)-1 and -2 have been shown to stimulate androstenedione production by human theca cells *in vitro*, and inhibin, which is induced by FSH and produced only in the granulosa cells, also induces thecal androgen synthesis [19]. The effect of inhibin is thought to represent an FSH-driven paracrine signal from the granulosa to the theca cells, affecting either the expression or the activity of CYP17, and thereby increasing androgen production. The effect of inhibin and IGF on androgen production is further enhanced by the addition of the action of LH on thecal cells [19].

A number of other factors have been shown to inhibit 17-hydroxypregnenolone, DHEA and androgen production by theca cells (i.e. CYP17 activity). These include epidermal growth factor (EGF), fibroblast growth factor (FGF), TGF-b, growth differentiation factor-9 and activin [8, 20–23]. The relevance of these various factors to the effects of FSH and LH on the steroid output of the follicle is yet to be determined.

#### Potential implications of CYP17 regulation for COS

It is not currently understood how the regulation of CYP17 enzyme activity in human theca cells is affected during COS, and whether this alters steroidogenesis compared with the normal menstrual cycle. It is possible that, for example, changes in CYP17 activity could affect the relative contribution of theca and granulosa cells to progesterone production. It seems likely that changes in CYP17 activity would affect the types of steroids produced by the theca cells because, as metabolism of pregnenolone via the D5 pathway to estradiol increased, so progesterone production would be expected to be reduced due to depletion of pregnenolone substrate for 3β-HSD. Currently, however, it is not known what proportion of the progesterone produced during the follicular phase is contributed by the theca and granulosa cells. Indeed, it is likely that the proportion of granulosa cell-derived progesterone will evolve as the follicle grows and the granulosa cells become more sensitive to LH. Given that CYP17 expression can be enhanced both by stimulation with LH and through paracrine mechanisms from the follicle (inhibin, via FSH), profound induction of CYP17 during COS may lead to a change in the substrate preference of the enzyme, or may be affected by an abundance of 17-hydroxyprogesterone.

#### Regulation of other enzymes

##### CYP11A

The first step in the process of steroidogenesis, the conversion of cholesterol to pregnenolone, is traditionally considered to be rate-limiting [11] for all further steroid metabolism. Chronic regulation is principally at the level of transcription of the gene for CYP11A (P450scc), the enzyme responsible for catalysing the conversion. Acute regulation is mediated by steroid acute regulatory protein (StAR), which facilitates the influx of cholesterol into the mitochondria, where the CYP11A enzyme is located. StAR expression is enhanced by cAMP and by stimulation of the granulosa cells with FSH and LH/human chorionic gonadotropin (hCG).

##### *3*β*-HSD*

The dehydrogenase enzyme 3β-HSD is responsible for catalysing the conversion of D5 steroids to the D4 configuration (Figure 1), and is therefore essential for the biosynthesis of all classes of steroid hormones. The expression of 3β-HSD has an important role in both theca and granulosa cells. The relative levels of activity of 3β-HSD and CYP17 determine whether pregnenolone is metabolised primarily via the D5 pathway (leading to estrogen production) or the D4 pathway (leading to progesterone and 17-hydroxyprogesterone).

In cultured granulosa cells from immature rats, FSH was shown to increase 3β-HSD mRNA, protein and enzyme activity [24, 25]. hCG/LH has also been shown to increase 3β-HSD expression in human ovarian theca cells [26]. Thus, although 3β-HSD expression appears to be highly constitutive, it can be augmented by both FSH in the granulosa cells and LH in the theca cells. Currently it is not known to what extent the activity of the enzyme can be up-regulated, or whether gonadotropins have differential effects on 3β-HSD activity in theca and granulosa cells, where such effects could have a major impact on follicular steroid output.

#### Human follicular fluid steroids

Analyses of steroid concentrations in human follicular fluids during the normal follicular phase appear to reflect this level of understanding of enzyme activity, and the interplay between the two cell types. It has been clearly demonstrated that high concentrations of androgens are universal in human follicles, indicating that thecal cell activity is intrinsic to steroidogenesis at all stages, irrespective of the source of progesterone. Furthermore, as follicles grow there is a general tendency for the relative proportion of steroids to evolve. The general pattern indicates that small follicles (FD <6 mm) are “androgenic” and larger follicles are “estrogenic” and highly “progestogenic” in pre-ovulatory follicles. The shift from the estrogenic state to the progestogenic state does not involve a reduction in the estrogen component, rather an increase in progesterone concentrations. This shift is consistent with both increased steroidogenesis by, and increased LH sensitivity of, granulosa cells [27].

### Source of progesterone during the follicular phase of COS and the implications for successful embryo implantation

Several studies have reported raised concentrations of progesterone in the late follicular phase of gonadotropin-releasing hormone (GnRH)-agonist and antagonist controlled COS (i.e. in the apparent absence of raised LH). There is continued debate regarding the clinical significance of these relatively modest concentration changes. It has been hypothesised that increases in circulating progesterone concentrations from non-luteinised tissues may lead to advances in the development of the endometrium and effectively reduce the window of implantation. As a result, excessive asynchrony between the development of the egg and the embryo, timed by the hCG or LH surge, and that of the endometrium, influenced by prematurely raised progesterone, would be likely to reduce implantation rates [28]. The concentration of progesterone that would be expected to have effects on the endometrium is likely to approximate that seen on the day after the LH surge during the follicular phase of a natural cycle. However, there is no reason to believe that elevated circulating progesterone affects the development of the egg or embryo, as the concentrations seen in each follicular fluid are many times higher than that seen in the circulation.

**Figure 3:**
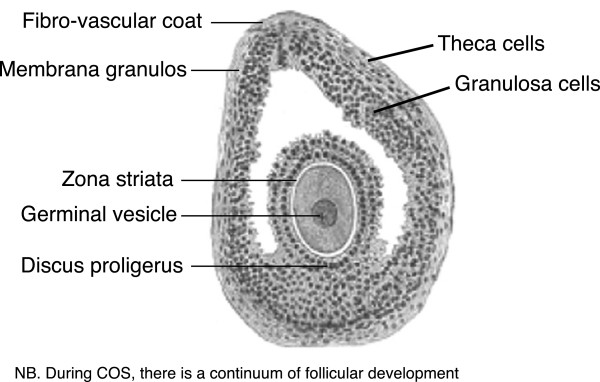
**The follicle as a factory of steroid biosynthesis.** During controlled ovarian stimulation, there is a continuum of follicular development. Source: http://en.wikipedia.org/wiki/Ovarian_follicle

It is critical to recognise that during COS, there is a continuum of follicular development (Figure 3). At any given timepoint, there will be a number of follicles at different stages of development – at least in young women with many growing follicles. Hormone concentrations measured in the peripheral circulation therefore depend on the contribution of multiple small, medium and large follicles. While the conversion of cholesterol is the rate-limiting step in steroidogenesis, during the follicular phase, the LH drive for androgen biosynthesis in the theca cells is critical in determining the output of the follicle.

#### Local and systemic progesterone concentrations during normal cycles and in COS

**Figure 4:**
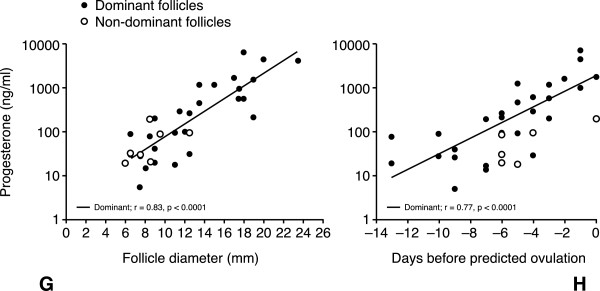
**Intrafollicular concentrations of progesterone in relation to follicular development in women during the follicular phase of the natural menstrual cycle.** Filled circles show the dominant follicles and open circles show the non-dominant follicles. Adapted from Schneyer AL et al. 2000 [29]*.*

The intrafollicular concentration of progesterone of women during the normal menstrual cycle was described by Schneyer and colleagues [29] who showed clearly the relationship between the production of progesterone and follicle diameter and the number of days before ovulation (Figure 4). Progesterone was detectable even in the smallest follicles and increased linearly with both follicle size (r = 0.83; p <0.0001) and maturity (r = 0.77; p <0.0001).

During the early follicular phase of a normal cycle, studies measuring steroid levels in ovarian veins showed that the estradiol concentration in veins draining the active follicles is already twice the maximum of that in the peripheral circulation; further increases are apparent by the mid-follicular phase [30]. Raised levels of progesterone can also be detected during the early follicular phase, while the follicles are still very small (10–12mm); by the mid-follicular phase, the progesterone concentration in the active veins is profoundly increased compared with the peripheral circulation [30]. Clearly, there is a tremendous output of progesterone by the developing follicle during normal cycles. While some of this enters the peripheral circulation, most seems to be metabolised by the liver such that peripheral concentrations are not increased prior to luteinisation.

During COS, the fact that multiple follicles are contributing to the steroid mix from both ovaries, progesterone output is likely to increase the progesterone level in the periphery. Exogenous FSH acting on the granulosa cells would be likely to increase circulating progesterone, whereas it is unclear whether LH drives the theca cells to produce androgens at the expense of progesterone. A positive correlation between LH surge and progesterone release has been reported [31].

However, in the early follicular phase, the granulosa cells do not respond well to low LH activity, and increases in LH (e.g. flare start) have been shown to increase progesterone output from what is considered to be the old corpus luteum. In a study by Cedrin-Durnerin, early increases of progesterone in response to LH increases were prevented in women who were pretreated with a progestogen (norethisterone) [32].

An analysis by Ubaldi et al. assessed whether premature luteinisation could occur in GnRH agonist and FSH (recombinant FSH and human urinary FSH) IVF cycles and whether premature luteinisation affected IVF and clinical outcome. Data indicated a positive correlation between FSH and progesterone levels, in that area under the FSH and progesterone curves were higher in cycles with premature luteinisation. Area under the FSH curve also correlated with the area under the progesterone curve, suggesting that increased FSH-induced LH receptivity in granulosa cells may be a factor in inducing premature luteinisation [33]. In a study by Beckers et al. in which investigators assessed luteal phase LH and progesterone concentrations without corpus luteum exogenous support, data also indicated concomitant increases in progesterone levels as FSH levels increased [34].

In the MERIT (menotropin versus recombinant FSH in-vitro fertilisation trial) study, where patients were undergoing a GnRH agonist long protocol, serum and follicular fluid progesterone concentrations were higher among those who received recombinant FSH (rFSH: GONAL-F) versus those treated with highly purified-hMG (MENOPUR). In contrast, the follicular fluid concentrations of androgens and oestrogen were higher in the hMG treated group. The raised circulating concentrations of progesterone were also related to the presence of a large follicular mass in patients treated with r-hFSH [35]. In a separate sub-analysis of MERIT, elevated circulating progesterone during the late follicular phase (>4nmol/L on the day of hCG administration) was associated with an increased ovarian response (egg yield) in both treatment groups [36]. The above data suggest that the higher incidence of elevated progesterone in the rFSH group was consistent with findings in this subgroup of a higher ovarian response, leading to more follicles collectively containing a higher mass of granulosa cells, a lower conversion of progesterone to androgens in the r-hFSH group (due to the lack of LH/hCG in rhFSH) and a modified endometrial receptivity due to advancement of endometrial development. The authors proposed a new and inexistent steroidogenic pathway in which the hCG contained in hMG can convert intrafollicular progesterone back to oestradiol.

Data from another study show that during treatment with a GnRH antagonist, a large reduction in circulating LH occurs for a short period of time (with no change in circulating FSH because exogenous FSH is administered), and that estradiol and progesterone concentrations are highly correlated with the level of LH [37].

**Figure 5:**
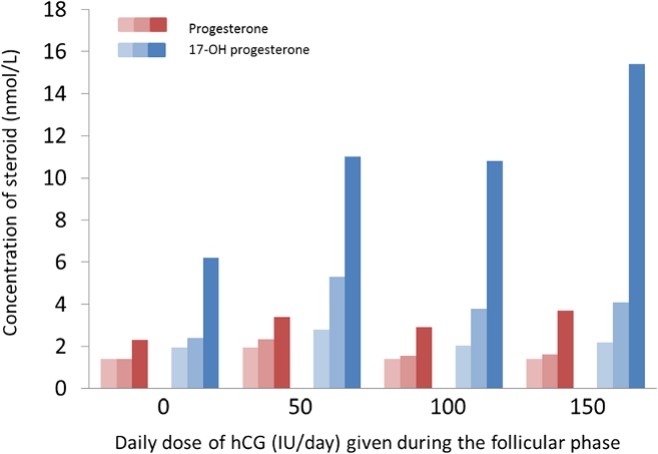
**Levels of progesterone and 17-OH-progesterone during the follicular phase in women stimulated with different doses of hCG.** Shaded bars represent stimulation day 0, day 6 and day of hCG triggering. *Graph drawn using data from Thuesen et al. 2013*[40]. hCG: human chorionic gonadotropin.

A further study of IVF/intracytoplasmic sperm injection (ICSI) patients using GnRH analogues for pituitary down-regulation [38] reported that elevated progesterone on the day of hCG administration was associated with a lower ongoing pregnancy rate. Patients with lower progesterone levels (≤1.5ng/mL) had significantly higher ongoing pregnancy rates than those with higher levels, irrespective of the GnRH analogue used for pituitary down-regulation. Achieving a higher ovarian response, as indicated by circulating oestradiol, appeared to be advantageous, in terms of achieving a higher ongoing pregnancy rate, only if progesterone was not elevated [38]. A well-conducted study by Thuesen et al. [39] sought to determine the optimal dose of hCG that would be compatible with good live birth rates. The authors examined serum progesterone levels in women treated with hCG at doses up to 150 IU from day 1 in combination with a fixed dose of 150 IU r-hFSH. A positive dose response in circulating progesterone was observed in this study but concentrations were considered to be below that at which impairment in the endometrium has been observed [39]. A later paper by the same group showed that the concentration of 17-OH progesterone in the late follicular phase of COS is much higher than that of progesterone. 17-OH progesterone does also act via the progesterone receptor and appears to be a more sensitive marker of premature increases in “progestin” activity than progesterone itself [40]. It is also possible that the relative concentration of progesterone and 17-OH progesterone in the follicular and luteal phase respectively is an important measure of progestin activity. In pre-ovulatory follicles there appears to be approximately four times more progesterone than 17-OH progesterone, while the study by Thuesen et al [40] showed that, in contrast, in the circulation, there is far more 17-OH progesterone than progesterone (Figure 5). This may be due to the conversion of progesterone to 17-OH progesterone upon leaving the follicle and passing through the theca cell layer. In the luteal phase there appear to be more equal concentrations of progesterone and 17-OH progesterone, but in this phase the secretion is directly into the bloodstream where progesterone is not readily converted to 17-OH progesterone.

The effect of elevated progesterone on pregnancy rates may be treatment dependent. A large multicentre randomised controlled trial of hMG versus rFSH in 749 women reported that in the rFSH group, women with progesterone levels >4nmol/L had a significantly ongoing lower pregnancy rate compared with those women with progesterone levels ≤4nmol/L. While in the hMG group, ongoing pregnancy rates were similar in women with progesterone levels ≤4nmol/L or >4 nmol/L [41]. However, a recent study comparing progesterone response to either r-hFSH/r-hLH or HP-hMG + urinary FSH reported that serum and intra-follicular progesterone levels and pregnancy rates were not significantly different between groups [42].

The elevation in progesterone following COS may also differ between low- and high-responder patients. In a study by Fatemi et al. [43] of women receiving either corifollitropin-α (an FSH agonist) or r-hFSH, elevated progesterone (>1.5 ng/ml) was observed more frequently in the high-responder groups in both treatment arms. However, this study concluded that a high ovarian response did not compromise ongoing pregnancy rates and increased cumulative pregnancy rates following fresh and frozen-thawed embryo transfer in patients treated with corifollitropin-α or rFSH [43]. A further study showed no detrimental effect of elevated progesterone in the late follicular phase of high-responder patients, but did observe an effect in low-responder patients [44]. This suggests that when a few follicles are stimulated to produce large amounts of progesterone this may compromise embryo quality rather than the endometrial receptivity and that the endometrium cannot distinguish between progesterone produced by a few or a large number of follicles. However, the lower pregnancy rates may reflect other reasons for the lower chance of becoming pregnant in these low-responder patients.

The Nordic LH Study Group conducted an analysis of progesterone measurements using archived blood samples from 526 women in a randomised, controlled trial that assessed rFSH + rLH (n = 265) versus rFSH alone (n = 261) in an agonist down-regulation long protocol for IVF/ICSI. The rFSH starting dose was 150 or 225 IU/day, respectively, in patients ≤35 and >35 years of age [45]. There were no significant differences between treatment groups with regard to oocyte retrieval, embryo transfer, implantation and ongoing clinical pregnancy rates. Circulating levels of LH were down-regulated on Days 1 and 6, and on the day of hCG administration, LH was significantly higher (p <0.001) in the rFSH + rLH group compared with the rFSH group [45]. Progesterone levels on Day 1 and on the day of ovulation induction were very similar between the treatment groups, and the progesterone concentration on the day of ovulation induction was significantly correlated with the number of oocytes retrieved and the number of follicles, but not with FSH consumption [46]. The level of LH on the day of ovulation induction was significantly correlated with the circulating progesterone level, but not with the number of oocytes. There was no significant relationship between biochemical or clinical pregnancy rates (per patient and per embryo transfer) and late follicular phase serum progesterone levels. In fact, the group of patients with very high progesterone levels (≥7nmol/L) had higher pregnancy rates than most of the other subgroups [46]. Of interest, a study examining the administration of DHEA in a woman with low ovarian reserve transplanted with cryopreserved ovarian tissue [47] showed an immediate augmentation of her progesterone levels. This suggests that the adrenal production of progesterone is important, especially with regard to low progesterone levels observed in the late follicular phase of the menstrual cycle.

These results indicate that the concentration of progesterone during the late follicular phase reflects the number of preovulatory follicles and oocytes retrieved, and that high circulating progesterone, may not have a deleterious effect on pregnancy outcomes. When looking at women in the early follicular phase, data also indicate a strong significant positive correlation between LH surge and progesterone release [31]. In vitro studies bovine follicular and luteal cells clearly showed that the higher the level of LH activity, the higher the production of progesterone [48].

It is important to note that commercial laboratory assays to measure progesterone levels were developed for use during the luteal phase (i.e. to determine whether or not a patient has ovulated), requiring a 5–10 fold higher sensitivity to progesterone levels than that which would occur during the follicular phase [49–51]. Therefore current assays that are used to test progesterone in the follicular phase of COS may yield imprecise values. Further, according to results of a survey of automated immunoassay analysers in Belgium, progesterone test results generally showed high inter-laboratory coefficients of variation (6–45%) and bias values. Investigators concluded that a linear relationship between the reference value and reported values was not assured for most testing methods from 0.56 to 117.85 nmol/L of progesterone [52].

## Conclusions

In this review, it has been shown that there is conflicting evidence on the effects of high progesterone levels (≥7 nmol/L) during the late follicular phase and the impact that this has on the establishment of pregnancy. In natural cycles, the concentration of progesterone in the follicular fluid at the time of the LH surge is very high compared with that of oestradiol [53], which indicates that high levels of progesterone may be a natural event at this time of the cycle. This demonstrates that high levels of progesterone are aligned with normal ovarian physiology, with high progesterone levels being required for the development of the follicle. The timing of the increase in progesterone levels is important for endometrial development, however, the final impact at the endometrial levels still needs to be clarified.

LH and FSH activity are important drivers of oestradiol, progesterone and androstenedione concentrations. During COS, increased progesterone is related to the increased number of follicles and thereby the total number of granulosa cells, and increased sensitivity to lower levels of LH, thus an increase in LH may induce a further limited increase in intrafollicular progesterone. In COS, the level of steroidogenesis per cell or per follicle does not increase, but total steroid production increases, in line with the increase in the number of growing follicles and cells.

In this article we have shown that cholesterol is the precursor for biosynthesis of steroids. Following the initial cleavage of cholesterol, the resulting pregnenolone molecule can be metabolised by either the D4 or D5 pathway. In the D4 pathway, the metabolism of pregnenolone to progesterone by 3β-HSD is an irreversible reaction, and progesterone can only be further metabolised to 17-hydroxyprogesterone. In the human ovary the only way to produce oestradiol is via the D5 pathway, where 17-hydroxypregnenolone can undergo further side-chain cleavage and modification. It is not currently known how the pathway that pregnenolone follows is determined or why 17-hydroxyprogesterone is the final product of the D4 pathway. It is also not currently known whether conversion of 17-hydroxypregnenolone to 17-hydroxyprogesterone or androstenedione is regulated differently in follicles of different sizes. Furthermore, it is important to understand how the regulation of CYP17 enzyme activity in human theca cells is affected during COS, and whether this alters steroidogenesis compared with the normal menstrual cycle.

**Figure 6:**
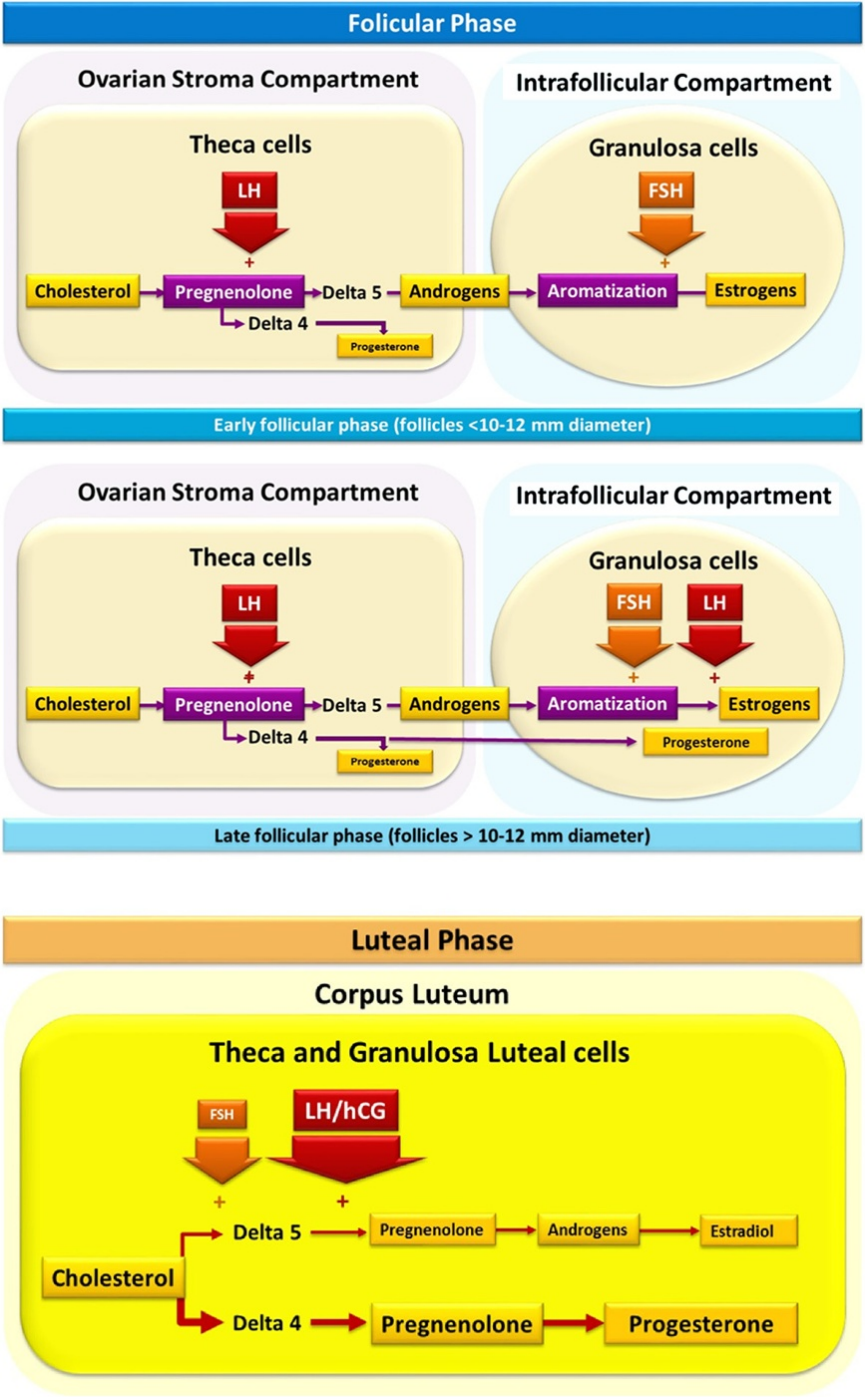
**Gonadotropic regulation of steroidogenesis at different phases of the ovarian cycle.** A summary of understanding based on the currently available literature reviewed in this article. FSH: follicule stimulating hormone; hCG: human chorionic gonadotropin; LH: luteinising hormone.

Based on the above evidence, it is not clear if the high levels of progesterone produced during COS have detrimental effects on fertility outcomes. High doses of FSH could be related to a reduced endometrial proliferation through direct or indirect actions. The MERIT and Nordic studies used high and low doses of FSH for the same population of patients, however, the studies show different results in relation to the high levels of progesterone and pregnancy outcomes [35, 45]. Based on this evidence it is possible that the differences are a reflection of FSH, progesterone or the combination of both acting at the endometrial level. Our current understanding of the regulation of steroidogenesis by gonadotropins at different stages of the ovarian cycle is outlined in Figure 6. However, unanswered questions remain and further research in this area is required to help elucidate the complex process of steroidogenesis and determine the future implications for COS.

## Abbreviations

ART: Assisted reproductive technology
cAMP: Cyclic adenosine monophosphate
COS: Controlled ovarian stimulation
DHEA: Dehydroepiandrosterone
EGF: Epidermal growth factor
FGF: Fibroblast growth factor
FSH: Follicle-stimulating hormone
GnRH: Gonadotropin-releasing hormone
hCG: Human chorionic gonadotropin
IGF: Insulin-like growth factor
IVF: In vitro fertilisation
LH: Luteinising hormone
MAP: Mitogen-activated protein (MAP)
POR: P450 oxidoreductase
rhFSH: Recombinant human FSH,
StAR: Steroid acute regulatory protein
TGF-b: Transforming growth factor beta.

## References

[1] Bourgain C, Devroey P (2003). The endometrium in stimulated cycles for IVF. Hum Reprod Update.

[2] Horcajadas JA, Mínguez P, Dopazo J, Esteban FJ, Domínguez F, Giudice LC, Pellicer A, Simón C (2008). Controlled ovarian stimulation induces a functional genomic delay of the endometrium with potential clinical implications. J Clin Endocrinol Metab.

[3] Sullivan MW, Stewart-Akers A, Krasnow JS, Berga SL, Zeleznik AJ (1999). Ovarian responses in women to recombinant follicle-stimulating hormone and luteinizing hormone (LH): a role for LH in the final stages of follicular maturation. J Clin Endocrinol Metab.

[4] Jeppesen JV, Kristensen SG, Nielsen ME, Humaidan P, Dal Canto M, Fadini R, Schmidt KT, Ernst E, Yding Andersen C (2012). LH-receptor gene expression in human granulosa and cumulus cells from antral and preovulatory follicles. J Clin Endocrinol Metab.

[5] Conti M (2002). Specificity of the cyclic adenosine 3′,5′-monophosphate signal in granulosa cell function. Biol Reprod.

[6] Tonetta SA, diZerega GS (1989). Intragonadal regulation of follicular maturation. Endocr Rev.

[7] Howles CM, Loumaye E, Giroud D, Luyet G (1994). Multiple follicular development and ovarian steroidogenesis following subcutaneous administration of a highly purified urinary FSH preparation in pituitary desensitized women undergoing IVF: a multicentre European phase III study. Hum Reprod.

[8] Filicori M, Cognigni GE, Taraborrelli S, Spettoli D, Ciampaglia W, de Fatis CT, Pocognoli P (1999). Luteinizing hormone activity supplementation enhances follicle-stimulating hormone efficacy and improves ovulation induction outcome. J Clin Endocrinol Metab.

[9] Fukuda S, Orisaka M, Tajima K, Hattori K, Kotsuji F (2009). Luteinizing hormone-induced Akt phosphorylation and androgen production are modulated by MAP Kinase in bovine theca cells. J Ovarian Res.

[10] Yong EL, Hillier SG, Turner M, Baird DT, Ng SC, Bongso A, Ratnam SS (1994). Differential regulation of cholesterol side-chain cleavage (P450scc) and aromatase (P450arom) enzyme mRNA expression by gonadotrophins and cyclic AMP in human granulosa cells. J Mol Endocrinol.

[11] Miller WL (2008). Steroidogenic enzymes. Endocr Dev.

[12] Wickenheisser JK, Nelson-DeGrave VL, McAllister JM (2006). Human ovarian theca cells in culture. Trends Endocrinol Metab.

[13] Smyth CD, Miró F, Whitelaw PF, Howles CM, Hillier SG (1993). Ovarian thecal/interstitial androgen synthesis is enhanced by a follicle-stimulating hormone-stimulated paracrine mechanism. Endocrinology.

[14] Whitelaw PF, Smyth CD, Howles CM, Hillier SG (1992). Cell-specific expression of aromatase and LH receptor mRNAs in rat ovary. J Mol Endocrinol.

[15] Young JM, McNeilly AS (2010). Theca: the forgotten cell of the ovarian follicle. Reproduction.

[16] Andersen CY (2002). Possible new mechanism of cortisol action in female reproductive organs: physiological implications of the free hormone hypothesis. J Endocrinol.

[17] Pandey AV, Miller WL (2005). Regulation of 17,20 lyase activity by cytochrome b5 and by serine phosphorylation of P450c17. J Biol Chem.

[18] Auchus RJ, Lee TC, Miller WL (1998). Cytochrome b5 augments the 17,20-lyase activity of human P450c17 without direct electron transfer. J Biol Chem.

[19] Nahum R, Thong KJ, Hillier SG (1995). Metabolic regulation of androgen production by human thecal cells in vitro. Hum Reprod.

[20] McAllister JM, Byrd W, Simpson ER (1994). The effects of growth factors and phorbol esters on steroid biosynthesis in isolated human theca interna and granulosa-lutein cells in long term culture. J Clin Endocrinol Metab.

[21] Spicer LJ, Aad PY, Allen DT, Mazerbourg S, Payne AH, Hsueh AJ (2008). Growth differentiation factor 9 (GDF9) stimulates proliferation and inhibits steroidogenesis by bovine theca cells: influence of follicle size on responses to GDF9. Biol Reprod.

[22] Fournet N, Weitsman SR, Zachow RJ, Magoffin DA (1996). Transforming growth factor-beta inhibits ovarian 17 alpha-hydroxylase activity by a direct noncompetitive mechanism. Endocrinology.

[23] Dooley CA, Attia GR, Rainey WE, Moore DR, Carr BR (2000). Bone morphogenetic protein inhibits ovarian androgen production. J Clin Endocrinol Metab.

[24] DeMoura MD, Choi D, Adashi EY (1997). Insulin-like growth factor-I-mediated amplification of follicle-stimulating hormone-supported progesterone accumulation by cultured rat granulosa cells: enhancement of steroidogenic enzyme activity and expression. Biol Reprod.

[25] Eimerl S, Orly J (2002). Regulation of steroidogenic genes by insulin-like growth factor-1 and follicle-stimulating hormone: differential responses of cytochrome P450 side-chain cleavage, steroidogenic acute regulatory protein, and 3beta-hydroxysteroid dehydrogenase/isomerase in rat granulosa cells. Biol Reprod.

[26] Payne AH, Hales DB (2004). Overview of steroidogenic enzymes in the pathway from cholesterol to active steroid hormones. Endocr Rev.

[27] Westergaard L, Christensen IJ, McNatty KP (1986). Steroid levels in ovarian follicular fluid related to follicle size and health status during the normal menstrual cycle in women. Hum Reprod.

[28] Fleming R, Jenkins J (2010). The source and implications of progesterone rise during the follicular phase of assisted reproduction cycles. Reprod Biomed Online.

[29] Schneyer AL, Fujiwara T, Fox J, Welt CK, Adams J, Messerlian GM, Taylor AE (2000). Dynamic changes in the intrafollicular inhibin/activin/follistatin axis during human follicular development: relationship to circulating hormone concentrations. J Clin Endocrinol Metab.

[30] Coutts JRT (1981). Functional morphology of the human ovary.

[31] Bungum L, Jacobsson AK, Rosén F, Becker C, Yding Andersen C, Güner N, Giwercman A (2011). Circadian variation in concentration of anti-Müllerian hormone in regularly menstruating females: relation to age, gonadotrophin and sex steroid levels. Hum Reprod.

[32] Cédrin-Durnerin I, Bulwa S, Hervé F, Martin-Pont B, Uzan M, Hugues JN (1996). The hormonal flare-up following gonadotrophin-releasing hormone agonist administration is influenced by a progestogen pretreatment. Hum Reprod.

[33] Ubaldi F, Camus M, Smitz J, Bennink HC, Van Steirteghem A, Devroey P (1996). Premature luteinization in in vitro fertilization cycles using gonadotropin-releasing hormone agonist (GnRH-a) and recombinant follicle-stimulating hormone (FSH) and GnRH-a and urinary FSH. Fertil Steril.

[34] Beckers NG, Laven JS, Eijkemans MJ, Fauser BC (2000). Follicular and luteal phase characteristics following early cessation of gonadotrophin-releasing hormone agonist during ovarian stimulation for in-vitro fertilization. Hum Reprod.

[35] Smitz J, Andersen AN, Devroey P, Arce JC (2007). MERIT Group: **Endocrine profile in serum and follicular fluid differs after ovarian stimulation with HP-hMG or recombinant FSH in IVF patients**. Hum Reprod.

[36] Andersen AN, Devroey P, Arce JC (2006). Clinical outcome following stimulation with highly purified hMG or recombinant FSH in patients undergoing IVF: a randomized assessor-blind controlled trial. Hum Reprod.

[37] Out HJ, Rutherford A, Fleming R, Tay CC, Trew G, Ledger W, Cahill D (2004). A randomized, double-blind, multicentre clinical trial comparing starting doses of 150 and 200 IU of recombinant FSH in women treated with the GnRH antagonist ganirelix for assisted reproduction. Hum Reprod.

[38] Bosch E, Labarta E, Crespo J, Simón C, Remohí J, Jenkins J, Pellicer A (2010). Circulating progesterone levels and ongoing pregnancy rates in controlled ovarian stimulation cycles for in vitro fertilization: analysis of over 4000 cycles. Hum Reprod.

[39] Thuesen LL, Loft A, Egeberg AN, Smitz J, Petersen JH, Nyboe Andersen A (2012). A randomized controlled dose–response pilot study of addition of hCG to recombinant FSH during controlled ovarian stimulation for in vitro fertilization. Hum Reprod.

[40] Thuesen LL, Smitz J, Loft A, Nyboe Andersen A (2013). Endocrine effects of hCG supplementation to recombinant FSH throughout controlled ovarian stimulation for IVF: a dose-response study. Clin Endocrinol (Oxf).

[41] Devroey P, Pellicer A, Andersen AN, Arce JC, On behalf of the Menopur in GnRH Antagonist Cycles with Single Embryo Transfer (MEGASET) Trial Group (2012). A randomized assessor-blind trial comparing highly purified hMG and recombinant FSH in a GnRH antagonist cycle with compulsory single-blastocyst transfer. Fertil Steril.

[42] Requena A, Cruz M, Ruiz FJ, Garcia-Velasco JA (2014). Endocrine profile following stimulation with recombinant follicle stimulating hormone and luteinizing hormone versus highly purified human menopausal gonadotropin. Reprod Biol Endocrinol.

[43] Fatemi HM, Doody K, Griesinger G, Witjes H, Mannaerts B (2013). High ovarian response does not jeopardize ongoing pregnancy rates and increases cumulative pregnancy rates in a GnRH-antagonist protocol. Hum Reprod.

[44] Griesinger G, Mannaerts B, Andersen CY, Witjes H, Kolibianakis EM, Gordon K (2013). Progesterone elevation does not compromise pregnancy rates in high responders: a pooled analysis of in vitro fertilization patients treated with recombinant follicle-stimulating hormone/gonadotropin-releasing hormone antagonist in six trials. Fertil Steril.

[45] Nyboeandersen A, Humaidan P, Fried G, Hausken J, Antila L, Bangsbøll S, Rasmussen PE, Lindenberg S, Bredkjaer HE, Meinertz H, Nordic LH study group (2008). Recombinant LH supplementation to recombinant FSH during the final days of controlled ovarian stimulation for in vitro fertilization. A multicentre, prospective, randomized, controlled trial. Hum Reprod.

[46] Andersen CY, Bungum L, Andersen AN, Andersen CY (2011). High late follicular phase serum progesterone levels during controlled ovarian stimulation have no impact on pregnancy rates and are not influenced by exogenous LH. Reprod Biomed Online.

[47] Strauss S, Greve T, Ernst E, Fraidakis M, Grudzinskas JG, Andersen CY (2014). Administration of DHEA augments progesterone production in a woman with low ovarian reserve being transplanted with cryopreserved ovarian tissue. J Assist Reprod Genet.

[48] Wolfenson D, Sonego H, Shaham-Albalancy A, Shpirer Y, Meidan R (1999). Comparison of the steroidogenic capacity of bovine follicular and luteal cells, and corpora lutea originating from dominant follicles of the first or second follicular wave. J Reprod Fertil.

[49] Fleming R (2008). Progesterone elevation on the day of hCG: methodological issues. Hum Reprod Update.

[50] Venetis CA, Kolibianakis EM, Papanikolaou E, Bontis J, Devroey P, Tarlatzis BC (2007). Is progesterone elevation on the day of human chorionic gonadotrophin administration associated with the probability of pregnancy in in vitro fertilization? A systematic review and meta-analysis. Hum Reprod Update.

[51] Bosch E (2008). Comment on: is progesterone elevation on the day of human chorionic gonadotrophin administration associated with the probability of pregnancy in in vitro fertilization? A systematic review and meta-analysis. By Venetis et al. (2007). Hum Reprod Update.

[52] Coucke W, Devleeschouwer N, Libeer JC, Schiettecatte J, Martin M, Smitz J (2007). Accuracy and reproducibility of automated estradiol-17beta and progesterone assays using native serum samples: results obtained in the Belgian external assessment scheme. Hum Reprod.

[53] Andersen CY, Morineau G, Fukuda M, Westergaard LG, Ingerslev HJ, Fiet J, Byskov AG (1999). Assessment of the follicular cortisol: cortisone ratio. Hum Reprod.

